# Effect of extending the period from oral administration of 5-aminolevulinic acid hydrochloride to photodynamic diagnosis during transurethral resection for non-muscle invasive bladder cancer on diagnostic accuracy and safety: a single-arm multicenter phase III trial

**DOI:** 10.1007/s10147-024-02638-5

**Published:** 2024-10-07

**Authors:** Rikiya Taoka, Hideo Fukuhara, Makito Miyake, Keita Kobayashi, Atsushi Ikeda, Kent Kanao, Yoshinobu Komai, Ryo Fujiwara, Yusuke Sato, Mikio Sugimoto, Toyonori Tsuzuki, Kiyohide Fujimoto, Keiji Inoue, Mototsugu Oya

**Affiliations:** 1 Department of Urology, Kagawa University Hospital, 1750-1, Ikenobe, Miki-Cho, Kita-Gun, Kagawa 761-0793 Japan; 2 Department of Urology, Kochi Medical School Hospital, Kochi, Japan; 3 Department of Urology, Nara Medical University Hospital, Kashihara, Nara Japan; 4 Department of Urology, Yamaguchi University Hospital, Ube, Yamaguchi Japan; 5 Department of Urology, University of Tsukuba Hospital, Tsukuba, Ibaraki Japan; 6 Department of Uro-Oncology, Saitama Medical University International Medicine Center, Hidaka, Saitama Japan; 7 Department of Urology, Cancer Institute Hospital, Japanese Foundation for Cancer Research, Tokyo, Japan; 8 Department of Urology, The University of Tokyo Hospital, Bunkyo, Tokyo Japan; 9 Department of Surgical Pathology, Aichi Medical University Hospital, Nagakute, Aichi Japan; 10 Center for Photodynamic Medicine, Kochi Medical School, Kochi University, Kochi, Japan; 11 Department of Urology, Keio University Hospital, Shinjuku, Tokyo Japan

**Keywords:** Non-muscle invasive bladder cancer, Photodynamic diagnosis, 5-Aminolevulinic acid, Multicenter single-arm phase III trial

## Abstract

**Background:**

In Japan, the authorized period (2–4 h) between oral administration of 5-aminolevulinic acid hydrochloride (5-ALA) and transurethral resection for non-muscle invasive bladder cancer (NMIBC) may restrict photodynamic diagnosis (PDD) usage. Therefore, this prospective, single-arm, phase III study aimed to evaluate the diagnostic accuracy and safety of PDD at an extended administration period (4–8 h).

**Methods:**

From January 2022 to May 2023, 161 patients with NMIBC were enrolled from eight hospitals. The primary endpoint was the blue light (BL) sensitivity of pathologically positive biopsies. The secondary endpoints were a comparison of the specificity and positive and negative prediction rates under BL and white light (WL) conditions.

**Results:**

A total of 1242 specimens comprising 337 histological NMIBC specimens were analyzed. BL-sensitivity was 95.3%. Its lower limit of 95% confidence interval (92.4–97.3%) exceeded the threshold (70%) of non-inferiority to authorized usage. Sensitivity and specificity were significantly higher and lower for BL than those for WL (95.3% vs. 61.1%, *P* < 0.001; 52.7% vs. 95.2%, *P* < 0.001), respectively. The positive and negative predictive rates were significantly lower and higher for BL than those for WL (42.9% vs. 82.7%, *P* < 0.001; 96.8% vs. 86.8%, *P* < 0.001), respectively. Of the 145 patients receiving 5-ALA, 136 (93.8%) and 75 (51.7%) experienced 377 adverse events and 95 adverse reactions, respectively, most of which were grade 1 or 2.

**Conclusion:**

For extended period, the efficacy of PDD for NMIBC was similar to that of authorized period, in terms of higher sensitivity and lower specificity compared with WL, and the safety was acceptable.

**Supplementary Information:**

The online version contains supplementary material available at 10.1007/s10147-024-02638-5.

## Introduction

Bladder cancer is the 9th most common cancer worldwide, with an estimated 613,791 new cases in 2022 (GLOBOCAN) [1]. Non-muscle invasive bladder cancer (NMIBC) accounts for approximately 75% of newly diagnosed bladder cancer cases [2]. In clinical practice, the patients with NMIBC are initially treated with transurethral resection of bladder tumor (TURBT) [3–5]. However, NMIBC recurs in approximately 50% of the patients and invades the muscle layer in 15–30% of patients during recurrence [2].

Like T1 and high-grade tumors, urothelial carcinoma in situ (CIS) is a major risk factor for recurrence and progression in NMIBC [3–5]. However, CIS is frequently undetectable via conventional white light (WL) cystoscopy; resulting in an estimated 10–30% of CIS cases being overlooked, leading to recurrence or progression of NMIBC [6, 7]. Photodynamic diagnosis (PDD) is recommended by the clinical practice guidelines in Europe, America, and Japan because it can address this unmet need for NMIBC [3–5].

A protoporphyrin IX precursor, 5-aminolevulinic acid hydrochloride (5-ALA HCl, hereafter 5-ALA), has recently garnered substantial interest as a new-generation photosensitive substance for PDD because PDD using orally administered 5-ALA significantly improves the diagnostic accuracy for NMIBC, especially for CIS, compared to WL-cystoscopy [8–10]. Moreover, TURBT using PDD (PDD-TURBT) significantly prolonged the recurrence-free survival of patients with NMIBC [8] than that using only WL. Furthermore, PDD-TURBT can reduce risks of progression and repeated recurrence and is potentially more cost-effective than WL-TURBT in patients with NMIBC [11–13].

In Japan, oral administration of 5-ALA is currently authorized 2–4 h before PDD-TURBT. However, within the authorized period, PDD-TURBT at the allotted time was often difficult to perform. Hence, TURBT without PDD may be preferred. To avoid such an evidence-practice gap, extending the administration period is a clinical need. Although no prospective studies have been reported, one retrospective study suggested that PDD has sufficient diagnostic accuracy and acceptable safety, even after > 4 h of 5-ALA exposure before TURBT [14]. Therefore, we hypothesized that PDD-TURBT performed 2–4 h and > 4 h after the oral administration of 5-ALA would show similar diagnostic accuracy and safety. Based on the clinical need and our hypothesis, we conducted this phase III study, SPP2C102 trial, to evaluate the diagnostic accuracy and safety of PDD performed > 4 h after the oral administration of 5-ALA in patients with NMIBC.

## Patients and methods

### Study objectives and design

The aim of this phase III study was evaluation of the diagnostic efficacy of PDD at the extended administration period (4–8 h before TURBT) rather than comparison of diagnostic efficacy at the extended period with that at the authorized administration period (2–4 h before TURBT). The sensitivity under blue light condition (BL-sensitivity) at the authorized period was shown to be similar to two previous studies (SPP2C101, 79.6% [95% confidence interval [CI]: 72.9–85.2%]; ALA-BC-1, 75.8% [95%CI: 67.3–83.0%]) [9, 10]. Therefore, we aimed to demonstrate the non-inferiority of BL-sensitivity at the extended administration period by examining whether the lower limit of the 95%CI of BL-sensitivity exceeded the threshold of 70%, which was based on the lower limits of 95%CI of BL-sensitivity (SPP2C101, 72.9%; ALA-BC-1, 67.3%) observed in previous studies and set this as the primary endpoint. As the superiority of BL-sensitivity over WL-sensitivity has clinical significance, a secondary endpoint was comparison of sensitivity between BL and WL. Specificity and positive and negative predictive rates were other secondary endpoints.

This single-arm, phase III, multicenter trial was registered as jRCT2061210055 and approved by the review board of each institution. In addition, this study was conducted in compliance with Good Clinical Practice (GCP) guidelines and the Declaration of Helsinki. The quality of the trials was controlled by monitoring data management.

After obtaining written consent, patients aged 20–85 years with primary or recurrent NMIBC who were ascertained to meet eligible criteria (Table 1) by the investigators of each institution were enrolled at the screening stage. The eligibility criteria were the same as those in previous studies [9, 10]; however, patients with low blood pressure (systolic blood pressure ≤ 100 mmHg or diastolic blood pressure ≤ 60 mmHg) and those with a history of hypotension during TURBT were excluded to ensure safety. To avoid interference with safety, antihypertensives were prohibited from the day before administration until before TURBT.

**Table 1:** Inclusion and exclusion criteria for patient enrollment

Inclusion criteria	
1.	Indication for TURBT.
2.	Ability to provide written consent and compliance with the schedule of hospital visits and the study protocol.
3.	Age between 20 and 85 years (at the time of providing consent).
4.	Eastern Cooperative Oncology Group performance status of 0–1.
5.	Clinical test values meet the following criteria (within 28 days before administration).
6.	Renal function: serum creatinine level < 1.5 times the upper limit of reference value.
7.	Liver function: AST, ALT (GPT), and serum bilirubin levels < 1.5 times the upper limit of reference values.
8.	Hematology: Platelet count ≥ 100,000/mm^3^.
9.	Suspected with newly diagnosed or recurrent NMIBC. In case of recurrence, > 90 days should have elapsed since the last treatment for NMIBC.

Exclusion criteria	
1.	History or complications of any of the following: Myocardial infarction, congestive heart failure, angina requiring treatment, arrhythmia requiring treatment.
2.	Serious complications, severe infection (contains active tuberculosis), active multiple cancers.
3.	Current or previous hypersensitivity to porphyrin analogs.
4.	Taking any other investigational drug within 90 days before screening for this clinical trial.
5.	Scheduled to participate in clinical research during the clinical trial period.
6.	Systolic blood pressure ≤ 100 mmHg or diastolic auto-cblood pressure ≤ 60 mmHg in the screening test.
7.	Pregnant/planning for pregnancy/lactating women. No intention of contraception.
8.	Ineligibility judged by the principal or sub-investigator due to other reasons.

*TURBT* transurethral resection of bladder tumor, *NMIBC* non-muscle invasive bladder cancer, *ALT* alanine aminotransferase, *AST* aspartate aminotransferase, *GPT* glutamic pyruvic transaminase

To estimate the sample size, we assumed the BL-sensitivity to be 78%, based on previous studies (79.6%, SPP2C101; 75.8%, ALA-BC-1) [9, 10]. In total, 313 pathologically positive biopsies would be required, considering a comparison using a test of the population proportion with the threshold (70%), a two-sided significance level of 5%, and a power of 90%. As 3.0 pathologically positives per patient (181/60 patients) were collected in the SPP2C101 study [9], 105 patients were estimated to obtain 313 pathologically positives in this study. To reduce the bias of the administration time, 106 patients were randomly assigned equally (*N* = 53) to the two strata with different ranges of administration period: stratum-1 (> 4– ≤ 6 h before TURBT) and stratum-2 (> 6– ≤ 8 h before TURBT). At enrollment, the eligible patients were randomly assigned via electronic data capture using a permuted block method with a predetermined block size (4 cases) to ensure an equal number of patients in each stratum. The size and the sequence of blocks were determined by the block allocators of the SBI before starting and were not disclosed until the end of study except to the allocators.

### Procedures

The investigational agent, SPP-005, a powder of 5-ALA (SBI Pharmaceuticals), was dissolved in water and orally administered at a dose of 20 mg/kg body weight according to the assigned stratum dosing time, as described above. Before resecting NMIBCs, one or two specimens were collected from each of the eight regions within the bladder by performing the following procedures for each region: If a WL-positive area was found, one biopsy was sampled from the area after confirming the presence or absence of red fluorescence under BL, which corresponding to BL-positive or BL-negative, respectively. If no WL-positive area was found, one biopsy was sampled from a BL-positive but WL-negative area. When no test-positive area was identified under both WL and BL, one negative biopsy was sampled from any area. Information regarding each biopsy was recorded.

The patients and investigators were not blinded because this was a single-arm study. However, pathological evaluation was performed in a blinded manner at a central pathology evaluation organization and the results were not disclosed to other participants until the database was locked. The appropriateness of the sampling procedure was confirmed by the Central Video Judgment Committee by blinding the stratum information.

### Statistical analysis

The observation period was 14 days after administration of SPP-005. For efficacy, main analysis and a secondary analysis for sensitivity were performed using the full analysis set (FAS) and per-protocol set (PPS), respectively. Safety was analyzed using the safety analysis set (SAS), which included all patients who received SPP-005. All adverse events (AEs) within 14 days of administration were encoded using MedDRA/J version 26.0. The McNemar test or chi-square test was used to test the significance of the differences. The Clopper–Pearson method was used to calculate the 95% CIs. Statistical analyses were performed using SAS software (version 9.4) for Microsoft Windows 10 or higher. *P* < 0.05 was considered statistically significant.

## Results

### Patients and samples

**Fig. 1:**
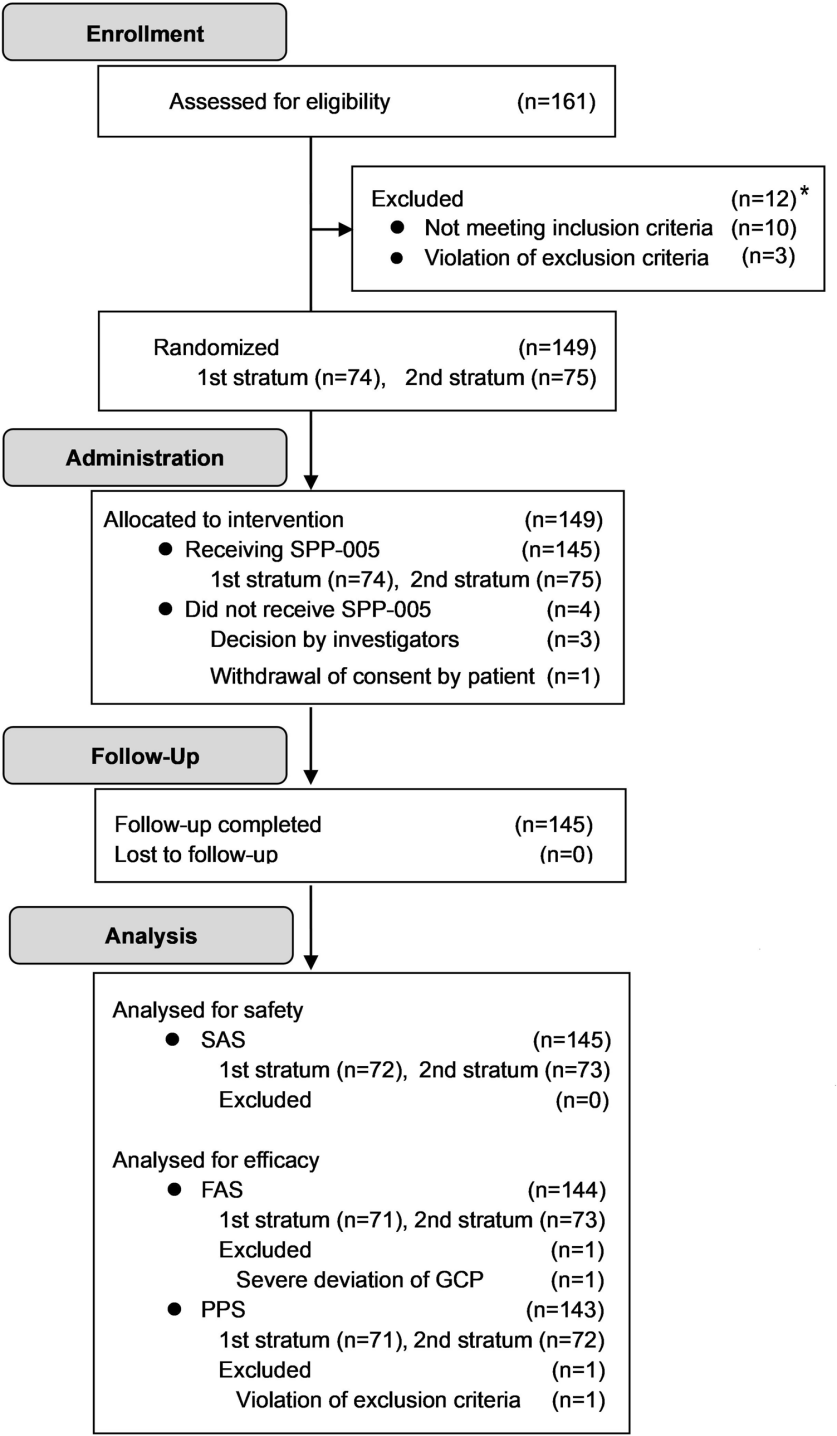
CONSORT flow diagram of patient enrollment. *SPP-005* 5-aminolevulinic acid hydrochloride powder, *SAS* safety analysis set, *FAS* full analysis set, *GCP* good clinical practice, *PPS* per-protocol set. *One patient was deemed ineligible based on the inclusion and exclusion criteria

Figure 1 shows a flow diagram of patient enrollment and data analysis. Between January 2022 and May 2023, 161 patients were enrolled because 313 positive specimens could not be obtained from 105 patients as planned before the start of the study. Of the 161 patients, 12 were excluded because of ineligibility and the remaining 149 were randomized (stratum-1, *N* = 74; stratum-2, *N* = 75). Among the 149 patients, 145 (stratum-1, *N* = 72; stratum-2, *N* = 73) who were administered SPP-005 completed the study and were included in the SAS. For the efficacy analysis, one case each was excluded from the FAS and PPS owing to GCP guideline deviation and exclusion criteria violation, respectively.

Table 2 shows the patient demographic and other baseline characteristics. The values for these items were the same as those in a previous study (SPP2C101) and no bias was observed between the total SAS and each stratum. Table 3 summarizes the biopsy samples. Among 1249 specimens collected, 1242 were selected for efficacy analysis using the FAS, including 337 pathologically positives and 905 pathologically negatives. The remaining seven specimens were excluded due to procedural deviations and because they were pathologically undiagnosable. Notably, all 337 specimens were NMIBC, including 148 flat NMIBC (pTis and UD), 174 papillary NMIBC (pTa), and 15 T1 NMIBC.

**Table 2:** Patient demographic and baseline characteristics (SAS)

	Total (*N* = 145)	Stratum-1 (*N* = 72)	Stratum-2 (*N* = 73)
Sex			
Male *n* (%)	118 (81.4)	55 (76.4)	63 (86.3)
Female *n* (%)	27 (18.6)	17 (23.6)	10 (13.7)
Age (years)			
Mean (SD)	69.4 (9.8)	70.3 (8.8)	68.5 (10.7)
Med, min, max	71.0, 39, 84	71.0, 46, 83	71.0, 39, 84
20–64 *n* (%)	38 (26.2)	15 (20.8)	23 (31.5)
65–74 *n* (%)	56 (38.6)	31 (43.1)	25 (34.2)
75–84 *n* (%)	51 (35.2)	26 (36.1)	25 (34.2)
Height (cm)			
Mean (SD)	164.22 (8.84)	162.74 (8.67)	165.67 (8.82)
Med, min, max	164.40, 139.2, 184.4	162.85, 139.2, 184.4	165.80, 146.8, 184.1
Weight (kg)			
Mean (SD)	64.26 (12.66)	62.08 (13.65)	66.42 (11.29)
Med, min, max	62.50, 37.6, 109.7	59.30, 37.6, 109.7	64.40, 40.3, 96.1
< 50 *n* (%)	14 (9.7)	12 (16.7)	2 (2.7)
≥ 50, < 60 *n* (%)	47 (32.4)	26 (36.1)	21 (28.8)
≥ 60, < 70 *n* (%)	41 (28.3)	15 (20.8)	26 (35.6)
> 70 *n* (%)	43 (29.7)	19 (26.4)	24 (32.9)
BMI (kg/m^2^)			
Mean (SD)	23.705 (3.559)	23.257 (3.819)	24.147 (3.249)
Med, min, max	23.191, 16.56, 37.60	22.307, 16.60, 37.60	23.524, 16.56, 32.51
Diagnosis of non-muscle invasive bladder cancer			
Primary case *n* (%)	92 (63.4)	43 (59.7)	49 (67.1)
Recurrence case *n* (%)	53 (36.6)	29 (40.3)	24 (32.9)
ECOG PS			
0 *n* (%)	142 (97.9)	71 (98.6)	71 (97.3)
1 *n* (%)	3 (2.1)	1 (1.4)	2 (2.7)
History of other diseases			
No *n* (%)	79 (54.5)	38 (52.8)	41 (56.2)
Yes *n* (%)	66 (45.5)	34 (47.2)	32 (43.8)
Complications			
No *n* (%)	8 (5.5)	4 (5.6)	4 (5.5)
Yes *n* (%)	137 (94.5)	68 (94.4)	69 (94.5)
Body temperature (℃)			
Mean (SD)	36.42 (0.37)	36.45 (0.39)	36.39 (0.35)
Med, min, max	36.50, 35.0, 37.4	36.50, 35.0, 37.4	36.50, 35.1, 37.1
Pulse rate (pulse/min)			
Mean (SD)	76.9 (13.7)	77.0 (15.6)	76.8 (11.7)
Med, min, max	75.0, 42, 125	76.0, 42, 125	75.0, 51, 106
Systolic blood pressure (mmHg)			
Mean (SD)	135.1 (14.7)	135.8 (15.6)	134.4 (13.9)
Med, min, max	136.0, 104, 186	136.5, 104, 186	136.0, 106, 165
Diastolic blood pressure (mmHg)			
Mean (SD)	80.6 (9.9)	80.1 (9.3)	81.1 (10.6)
Med, min, max	80.0, 61, 108	81.0, 62, 98	80.0, 61, 108

*SD* standard deviation, *Med* median, *Min* minimum, *Max* maximum

**Table 3:** Summary of biopsy samples

	FAS (*N* = 144)	PPS (*N* = 143)
Total samples	1249	1242
Analyzed samples	1242	1212
Pathological positive	337	330
NMIBC	337	330
Flat NMIBC	148	146
Papillary NMIBC (pTa)	174	169
pT1	15	15
Pathological negative	905	882
Not analyzed	7	30

*FAS* full analysis set, *PPS* per protocol set, *NMIBC* non-muscle invasive bladder cancer

### Efficacy

Table 4 summarizes the efficacy of TURBT 4–8 h after 5-ALA administration. Regarding the primary, the lower limit of 95% CI of BL_-_sensitivity exceeded 70%, the threshold of non-inferiority; therefore, the outcome during the administration period in this study (at 4–8 h before TURBT) was not inferior to that of the authorized period (at 2–4 h before TURBT). In addition, the BL-sensitivity in both stratum-1 (95.5% [147/154, 95%CI: 90.9–98.2%]) and stratum-2 (95.1% [174/183, 95%CI: 90.9–97.7%]) (Supplemental Table 1) also exceeded the threshold. Furthermore, the BL-sensitivity of FAS total was significantly higher than WL_-_sensitivity (61.1% [206/337, 95%CI: 55.7–66.4%]) (*P* < 0.001, McNemar test). In contrast, BL-specificity was significantly lower than WL-specificity (52.7% vs. 95.2%, *P* < 0.001, McNemar test) and was similar in the FAS total and in each stratum (Supplemental Table 1). The positive predictive rate was significantly lower in BL than in WL (42.9% vs. 82.7%, *P* < 0.001, chi-squared test), whereas the negative predictive rate was significantly higher in BL than in WL (96.8% vs. 86.8%, *P* < 0.001, chi-squared test).

**Table 4:** Sensitivity, specificity, and positive and negative predictive rates (FAS)

Sensitivity							
Pathological positive	BL			WL			*P*-value
	Test		% (95% CI)	Test		% (95% CI)	
337	Positive	321	95.3 (92.4–97.3)	Positive	206	61.1 (55.7–66.4)	< 0.001^a^
	Negative	16		Negative	131		

Specificity							
Pathological negative	BL			WL			
	Test		% (95% CI)	Test		% (95% CI)	
905	Negative	477	52.7 (49.4–56.0)	Negative	862	95.2 (93.7–96.5)	< 0.001^a^
	Positive	428		Positive	43		

Positive predictive rate								
BL				WL				
Test positive	Pathology		% (95% CI)	Test positive	Pathology		% (95% CI)	
749	Positive	321	42.9 (39.3–46.5)	249	Positive	206	82.7 (77.5–87.2)	< 0.001^b^
	Negative	428			Negative	43		

Negative predictive rate								
BL				WL				
Test negative	Pathology			Test negative	Pathology			
493	Negative	477	96.8 (94.8–98.1)	993	Negative	862	86.8 (84.5–88.9)	< 0.001^b^
	Positive	16			Positive	131		

*FAS* full analysis set, *BL* blue light, *WL* white light, *CI* confidence interval

^a^McNemar test

^b^Chi-square test

Table 5 shows the sensitivity according to the tumor type. Precancerous urothelial dysplasia was included in NMIBC, as in the SPP2C101 study. The BL-sensitivity for flat NMIBC (93.9% [139/148, 95% CI: 88.8–97.2%]) was significantly higher than that under WL (26.4% [39/148, 95%CI: 19.5–34.2%]) (*P* < 0.001, McNemar test). The BL-sensitivity for papillary NMIBC (pTa) (96.6% [168/174, 95%CI: 92.6–98.7%]) was significantly higher than that under WL (91.4% [159/174, 95%CI: 86.2–95.1%]) (P = 0.020, McNemar test). The tumor detection rate by BL alone for all NMIBC (35.6% [120/337, 95%CI: 30.5–41.0%] was higher than that by WL (1.5% [5/337, 95%CI: 0.5–3.4%]) and was particularly high for flat NMIBC (68.2% [101/148, 95%CI: 60.1–75.6%]).

**Table 5:** Sensitivity and tumor detection rate only by blue light or white light in each tumor type (FAS)

Sensitivity by tumor-type						McNemar test
	Pathological positive	BL		Testpositive	WL	
		Test positive	Sensitivity (95% CI)		Sensitivity (95% CI)	*P*-value
NMIBC	337	321	95.3 (92.4–97.3)	206	61.1 (55.7– 66.4)	< 0.001
Flat NMIBC	148	139	93.9 (88.8–97.2)	39	26.4 (19.5–34.2)	< 0.001
UD	1	1	100.0 (2.5–100.0)	1	100.0 (2.5–100.0)	–
CIS	147	138	93.9 (88.7–97.2)	38	25.9 (19.0–33.7)	< 0.001
Papillary NMIBC (pTa)	174	168	96.6 (92.6–98.7)	159	91.4 (86.2–95.1)	0.020
NIPUCL	102	98	96.1 (90.3–98.9)	89	87.3 (79.2–93.0)	0.007
NIPUCH	72	70	97.2 (90.3–99.7)	70	97.2 (90.3–99.7)	1.000
pT1	15	14	93.3 (68.1–99.8)	8	53.3 (26.6–78.7)	0.034

Tumor detection rate only by BL or WL					
	Pathological positive	Positive only by BL		Positive only by WL	
		Sample	Rate (95% CI)	Sample	Rate (95% CI)
NMIBC	337	120	35.6 (30.5–41.0)	5	1.5 (0.5–3.4)
Flat NMIBC	148	101	68.2 (60.1–75.6)	1	0.7 (0.0–3.7)
UD*	1	0	0.0 (0.0–97.5)	0	0.0 (0.0–97.5)
CIS	147	101	68.7 (60.5–76.1)	1	0.7 (0.0–3.7)
Papillary					
NMIBC (pTa)	174	12	6.9 (3.6–11.7)	3	1.7 (0.4–5.0)
NIPUCL	102	10	9.8 (4.8–17.3)	1	1.0 (0.0–5.3)
NIPUCH	72	2	2.8 (0.3–9.7)	2	2.8 (0.3–9.7)
pT1	15	7	46.7 (21.3–73.4)	1	6.7 (0.2–31.9)

*FAS* full analysis set, *BL* blue light, *WL* white light, *CI* confidence interval, *NMIBC*, non-muscle invasive bladder cancer, *UD* urothelial dysplasia, *CIS* carcinoma in situ, *NIPUCL* non-invasive papillary urothelial cancer low-grade, *NIPUCH* non-invasive papillary urothelial cancer high-grade, *pT1* invasive urothelial tumor (pT1)

^*^For efficacy comparison, precancerous UD was included in NMIBC, as in the SPP2C101 study

### Safety

Of the 145 patients who were administered SPP-005, 377 AEs and 95 adverse drug reactions (ADRs) in 136 (93.8%) and 75 (51.7%) patients, respectively, were reported (Table 6). The frequency of AEs (stratum-1, 95.8%; stratum-2, 91.8%) and ADRs (stratum-1, 59.7%; stratum-2, 43.8%) was similar between the two strata. Most AEs and ADRs were grade 1 or 2. No deaths or discontinuations were reported. Five patients developed five serious AEs, including urinary retention in two patients and pyrexia, urinary tract infection, and hematuria in one patient each. Their causal relationship with the SPP-005 administration was ruled out. The ADRs noted in > 10% of the patients were hypotension (17.9% [26/145]) and hepatic function abnormal (11.7% [17/145]). No photosensitivity reactions were observed. All AEs were known nonserious events that occurred in the SPP2C101 study; therefore, no novel clinical concerns were considered.

**Table 6:** Summary of AEs and ADRs (SAS: *N* = 145)

	AEs		ADRs	
	Events	Cases (%)	Events	Cases (%)
All AEs				
All (*N* = 145)	377	136 (93.8)	95	75 (51.7)
Stratum-1 (*N* = 72)	203	69 (95.8)	57	43 (59.7)
Stratum-2 (*N* = 73)	174	67 (91.8)	38	32 (43.8)
Grade				
Grade 1	177	25 (17.2)	47	34 (23.4)
Grade 2	192	103 (71.0)	47	40 (27.6)
Grade 3	8	8 (5.5)	1	1 (0.7)
Grade ≥ 4	0	0	0	0
Discontinuation	0	0	0	0
Serious	5	5 (3.4)	0	0

SOC		
PT	ADRs	
	Events	Cases (%)
Vascular disorders	26	26 (17.9)
Hypotension	26	26 (17.9)
Investigations	24	20 (13.8)
Blood pressure decreased	10	10 (6.9)
Amylase increased	4	4 (2.8)
Alanine aminotransferase increased	4	4 (2.8)
Aspartate aminotransferase increased	3	3 (2.1)
Liver function test increased	1	1 (0.7)
Liver function test abnormal	1	1 (0.7)
Hepatic enzyme increased	1	1 (0.7)
Gastrointestinal disorders	22	21 (14.5)
Nausea	11	11 (7.6)
Vomiting	9	9 (6.2)
Constipation	2	2 (1.4)
Hepatobiliary disorders	19	19 (13.1)
Hepatic function abnormal	17	17 (11.7)
Liver disorder	2	2 (1.4)
Skin and subcutaneous tissue disorders	2	2 (1.4)
Rash	1	1 (0.7)
Dermatitis	1	1 (0.7)
Injury, poisoning, and procedural complications	1	1 (0.7)
Procedural hypotension	1	1 (0.7)
Respiratory, thoracic, and mediastinal disorders	1	1 (0.7)
Hiccups	1	1 (0.7)

MedDRA Ver 26.0

*SOC* systematic organ class, *PT* preferred term, *SAS* safety analysis set, *AEs* adverse events, *ADRs* adverse drug reactions

## Discussion

This phase III trial was designed based on the clinical need to extend the time from 5-ALA oral administration to the initiation of PDD-TURBT for NMIBC. As a result, BL-sensitivity for NMIBC 4–8 h after oral 5-ALA administration was 95.3%, which was significantly higher than WL-sensitivity (61.1%, *P* < 0.001). Particularly, BL-sensitivity for flat lesions, including CIS, was significantly higher than WL-sensitivity (93.9% vs. 26.4%, *P* < 0.001), with nonserious and acceptable AE rates. This result is similar to that of a previous study, wherein 5-ALA was administered 2–4 h before PDD and demonstrates the validity of 5-ALA oral administration 4–8 h before PDD in terms of safety and diagnostic accuracy for NMIBC.

In a recent systematic review, tumors remaining after the initial WL-TURBT were detected at the second transurethral resection (2nd-TUR) in 17–67% of patients following Ta and in 20–71% of patients following T1 cancer [15]. The primary performance requirement for PDD is high sensitivity because residual tumors can lead to early postoperative recurrence. In 2017, 5-ALA for PDD-TURBT was authorized and became available under Japan’s public health insurance system based on the results of two trials [9, 10]. The BL-sensitivities determined using 20 mg/kg body weight 5-ALA, which was orally administered 2–4 h before TURBT, were 75.8% and 79.6%, respectively, higher than the WL-sensitivity (47.6% and 54.1%, *P* < 0.001 and *P* < 0.001, respectively) [9, 10]. In our trial, BL-sensitivity (95.3%) at an extended administration time (4–8 h before TURBT) was higher than that at the authorized time (2–4 h before TURBT) in previous trials (79.6% and 75.8%) [9, 10]. One explanation for this discrepancy is improvements in the endoscopic camera system, as 2–4 K HD cameras were used in this trial instead of low-resolution (768 × 494 pixels) cameras in the previous trials. On the other hand, BL-specificity in this study (52.7%) was lower than that in previous studies (SPP2C101, 80.6%; ALA-BC-1, 68.2%) [9, 10]. Improvement in the camera system may be responsible for the lower specificity, as the detection of faint red fluorescence in normal urothelial mucosa may have resulted in an increase in false positive results. The increase in false-positives with a decrease in specificity is clinically acceptable, because the treatment strategy of NMIBC is based on pathological positivity.

In this trial, PDD had a lower specificity and positive predictive rate but a higher negative predictive rate than WL. This trend in diagnostic performance is similar to that in previous trials [9, 10], suggesting that PDD-TURBT was valid at the extended period as well as at the authorized period. The low positive predictive rate of PDD may increase false-positive resections. However, it may offer recurrence-preventing effects [16] because some false-positive specimens may contain hidden precancerous lesions leading to recurrence [17]. The significantly higher negative predictive rate under BL than under WL may be clinically significant regarding the reduction of unnecessary resections of non-cancerous tissues.

Flat lesions, such as CIS, are difficult to identify using WL-cystoscopy. However, owing to their strong association with recurrence [7, 8], their identification is essential to improve treatment outcomes in patients with NMIBC. This study revealed that the BL-sensitivity for flat lesions such as CIS was significantly higher than that of WL alone. In addition, the BL-sensitivity for T1 lesions was also significantly higher than that of WL alone, with 7 of 15 T1 lesions detected only under BL alone. High BL sensitivity for T1 lesions may contribute to improving the prognosis of patients with NMIBC because T1 lesions invading submucosa are high-risk factors that are frequently associated with the finding of residual tumor (50%) and upstaging to muscle-invasive disease (10%) at second TUR [18]. A Cochrane Review showed that PDD-TURBT may prevent the muscular progression of high-risk NMIBC, including CIS [19]. Thus, the results of this trial suggest that PDD-TURBT performed 4–8 h after oral administration of 5-ALA may reduce the risk of recurrence and progression through the diagnosis and treatment of CIS and other NMIBCs, including T1 lesions.

The incidence of AEs (93.8%) and ADRs (51.7%) in this study was comparable with those in the previous trials (AE: 95.1% [9], ADR: 45.9% [10]); therefore, the extension of the period between 5-ALA administration and PDD-TURBT in this study did not affect these incidence rates. The incidence of grade 3 ADR (0.7% [1/145 cases]) was lower than that in previous studies, and all ADRs were reversible, indicating that oral administration of 5-ALA 4–8 h before PDD is clinically acceptable. However, the incidence rate of hypotension-related ADRs (25.5%) in this study was higher than that in the previous trial (1.6%) [9]. Severe hypotension-related ADR was not reported in this study, whereas the incidence was 1.6% in the previous study [9]. In this study, the patients with lower blood pressure were excluded. In addition, the incidence of hypotension-related ADRs was announced at the time of this study [20–22]. The risk of hypotension-related ADRs itself may not change because these factors are thought to be the cause of the difference.

Nevertheless, a limitation of this trial was that the efficacy at different administration periods could not be directly compared since it was conducted as a single-arm, uncontrolled study. Thus, we set a threshold of non-inferiority to the authorized period (2–4 h) at 70%; however, the BL_-_sensitivity in this study was unexpectedly higher than that in previous studies, presumably due to factors such as the improvement of the camera system. Consequently, between-study comparison of BL-sensitivity was difficult; however, the trend of the diagnostic performance in this trial was similar to that in previous studies [9, 10]. Another limitation is that the criteria for PDD-positivity may vary among surgeons at different institutions. However, it can be corrected to some extent through two approaches: training of all surgeons to standardize PDD_-_positivity and central video evaluation of PDD_-_positivity. A short observation period was also a limitation of this study; therefore, the impact of the accurate diagnosis in improving postoperative prognosis remains to be examined, although the diagnostic accuracy and safety of ALA-PDD were demonstrated.

In conclusion, this trial demonstrated the validity of oral administration of 5-ALA 4–8 h before PDD by evaluating its efficacy and safety. Therefore, expansion of the administration period (2–8 h before TURBT) may increase the clinical use of ALA-PDD and ultimately improve the therapeutic effects of TURBT in patients with NMIBC.

## Supplementary Information

Below is the link to the electronic supplementary material.

Supplementary file1 (DOCX 25 KB)

## Data Availability

The data supporting the findings of this study are available within the paper or upon reasonable request for the corresponding author.
