# Correction to: Effect of extending the period from oral administration of 5-aminolevulinic acid hydrochloride to photodynamic diagnosis during transurethral resection for non-muscle invasive bladder cancer on diagnostic accuracy and safety: a single-arm multicenter phase III trial

**DOI:** 10.1007/s10147-024-02654-5

**Published:** 2024-11-19

**Authors:** Rikiya Taoka, Hideo Fukuhara, Makito Miyake, Keita Kobayashi, Atsushi Ikeda, Kent Kanao, Yoshinobu Komai, Ryo Fujiwara, Yusuke Sato, Mikio Sugimoto, Toyonori Tsuzuki, Kiyohide Fujimoto, Keiji Inoue, Mototsugu Oya

**Affiliations:** 1 Department of Urology, Kagawa University Hospital, 1750-1, Ikenobe, Miki-Cho, Kita-Gun, Kagawa 761-0793 Japan; 2 Department of Urology, Kochi Medical School Hospital, Kochi, Japan; 3 Department of Urology, Nara Medical University Hospital, Kashihara, Nara Japan; 4 Department of Urology, Yamaguchi University Hospital, Ube, Yamaguchi Japan; 5 Department of Urology, University of Tsukuba Hospital, Tsukuba, Ibaraki Japan; 6 Department of Uro-Oncology, Saitama Medical University International Medicine Center, Hidaka, Saitama Japan; 7 Department of Urology, Cancer Institute Hospital, Japanese Foundation for Cancer Research, Tokyo, Japan; 8 Department of Urology, The University of Tokyo Hospital, Bunkyo, Tokyo Japan; 9 Department of Surgical Pathology, Aichi Medical University Hospital, Nagakute, Aichi Japan; 10 Center for Photodynamic Medicine, Kochi Medical School, Kochi University, Kochi, Japan; 11 Department of Urology, Keio University Hospital, Shinjuku, Tokyo Japan

**Correction to: International Journal of Clinical Oncology** 10.1007/s10147-024-02638-5

The article Effect of extending the period from oral administration of 5-aminolevulinic acid hydrochloride to photodynamic diagnosis during transurethral resection for non-muscle invasive bladder cancer on diagnostic accuracy and safety: a single-arm multicenter phase III trial by Rikiya Taoka, Hideo Fukuhara, Makito Miyake, Keita Kobayashi, Atsushi Ikeda, Kent Kanao, Yoshinobu Komai, Ryo Fujiwara, Yusuke Sato, Mikio Sugimoto, Toyonori Tsuzuki, Kiyohide Fujimoto, Keiji Inoue and Mototsugu Oya was originally published electronically on the publisher’s internet portal https://link.springer.com/article/10.1007/s10147-024-02638-5 on 7 October 2024 without Open Access.

With the author(s)’ decision to opt for Open Choice, the copyright of the article changed on November 8, 2024 to © The Author(s) 2024 and the article is forthwith distributed under the terms of the Creative Commons Attribution 4.0 International License (https://creativecommons.org/licenses/by/4.0/), which permits use, duplication, adaptation, distribution and reproduction in any medium or format, as long as you give appropriate credit to the original author(s) and the source, provide a link to the Creative Commons license, and indicate if changes were made.

The original article has been corrected.

**Open Access** This article is distributed under the terms of the Creative Commons Attribution 4.0 International License (http://creativecommons.org/licenses/by/4.0/), which permits unrestricted use, distribution, and reproduction in any medium, provided you give appropriate credit to the original author(s) and the source, provide a link to the Creative Commons license, and indicate if changes were made. Publisher’s Note Springer Nature remains neutral with regard to jurisdictional claims in published maps and institutional affiliations.

