# Supplementary material for: Effect of extending the period from oral administration of 5-aminolevulinic acid hydrochloride to photodynamic diagnosis during transurethral resection for non-muscle invasive bladder cancer on diagnostic accuracy and safety: a single-arm multicenter phase III trial
Source: Int J Clin Oncol. 2024 Oct 7;30(1):110–20. doi: 10.1007/s10147-024-02638-5 (PMC11700051; doi:10.1007/s10147-024-02638-5)
Supplement: Supplementary file 1 — Supplementary file1 (DOCX 25 KB) [file 10147_2024_2638_MOESM1_ESM.docx]

Supplemental table 1. Sensitivity under BL in each stratum (FAS)

| BL-sensitivity | | | |
| --- | --- | --- | --- |
|  | Administration period | BL-positive / Pathological positive | 95% CI |
| FAS total | 4-8h before TURBT | 321 / 337 | 95.3% (92.4, 97.3) |
| Stratum-1 | 4-6h before TURBT | 147 / 154 | 95.5% (90.9, 98.2) |
| Stratum-2 | 6-8h before TURBT | 174 / 183 | 95.1% (90.9, 97.7) |
| BL-specificity | | | |
|  | Administration period | BL-negative / Pathological negative | 95% CI |
| FAS total | 4-8h before TURBT | 477 / 905 | 52.7% (49.4, 56.0) |
| Stratum-1 | 4-6h before TURBT | 237 / 450 | 52.7% (47.9, 57.4) |
| Stratum-2 | 6-8h before TURBT | 240 / 455 | 52.7% (48.0, 57.4) |

BL, Blue light; CI, Confidence interval
